# The CHRONOS Real-World Evidence of Biologic Treatments in Psoriatic Arthritis in Italy: A *Post Hoc* Gender Analysis

**DOI:** 10.1089/whr.2021.0063

**Published:** 2022-02-02

**Authors:** Delia Colombo, Micol Frassi, Giuseppa Pagano Mariano, Enrico Fusaro, Claudia Lomater, Patrizia Del Medico, Florenzo Iannone, Rosario Foti, Massimiliano Limonta, Antonio Marchesoni, Bernd Raffeiner, Ombretta Viapiana, Marco Di Carlo, Rosa Daniela Grembiale, Giuliana Guggino, Paola Faggioli, Enrico Tirri, Roberto Perricone, Pier Carlo Sarzi Puttini, Salvatore De Vita, Fabrizio Conti, Sara Rizzoli, Barbara Roncari, Martina Fiocchi, Roberto Orsenigo, Emanuela Zagni

**Affiliations:** ^1^ Novartis Farma S.p.A, Origgio, Italy.; ^2^ ASST Spedali Civili, Brescia, Italy.; ^3^ Grande Ospedale Metropolitano Bianchi Melacrino Morelli, Reggio Calabria, Italy.; ^4^ AOU Città della Salute e della Scienza di Torino, Turin, Italy.; ^5^ A.O. Mauriziano, Turin, Italy.; ^6^ Ospedale civile, Civitanova Marche, Italy.; ^7^ A.O.U. Policlinico Consorziale, Bari, Italy.; ^8^ A.O.U. Policlinico-Vittorio Emanuele, Catania, Italy.; ^9^ ASST Papa Giovanni XXIII, Bergamo, Italy.; ^10^ Department of Rheumatology, ASST Gaetano Pini-CTO, Milan, Italy.; ^11^ Ospedale Centrale di Bolzano, Bolzano, Italy.; ^12^ AOUI Verona Borgo Rome, Verona, Italy.; ^13^ Rheumatology Clinic, Università Politecnica delle Marche, Jesi (Ancona), Italy.; ^14^ A.O.U. Mater Domini, Catanzaro, Italy.; ^15^ A.O.U. Policlinico Giaccone, Palermo, Italy.; ^16^ Ospedale Civile di Legnano, Legnano, Italy.; ^17^ Ospedale San Giovanni Bosco, Naples, Italy.; ^18^ Policlinico Tor Vergata, Rome, Italy.; ^19^ ASST FBF Sacco, Milan, Italy.; ^20^ ASUIUD, Udine, Italy.; ^21^ Azienda Policlinico Umberto I, Rome, Italy.; ^22^ MediNeos Observational Research, Modena, Italy.

**Keywords:** biologics, effectiveness, gender differences, psoriatic arthritis, real-world evidence, observational study

## Abstract

***Background:*** Phenotypic features and outcome differences between sexes have been reported in psoriatic arthritis (PsA). However, little is known about sex differences in effectiveness of biologics in clinical practice.

***Methods:*** *Post hoc* gender analysis of the CHRONOS, a multicenter, noninterventional, retroprospective Italian real-world study assessing 6-month and 1-year effectiveness of biologics for PsA.

***Results:*** Eligible patients were 399, 43.1% men. Sociodemographic characteristics, type of arthritis, baseline Disease Activity Score 28 joints (DAS28), and duration of biologic treatment were rather homogeneous. More men were overweight/obese and naive to biologics. The most frequently used biologics were TNF-inhibitors and secukinumab in both sexes. DAS28 responders were 72.7% (women) and 70.5% (men) at 6 months, and 68.0% in both sexes at 1 year. American College of Rheumatology (ACR) response showed a trend for men versus women to achieve more frequently ACR50 (32.6% vs. 26.5% at 6 months; 34.9% vs. 20.0% at 1 year) and ACR70 (22.3% vs. 12.4% at 6 months and 25.0% vs. 13.0% at 1 year). Global satisfaction with treatment at enrollment and after 6 months was slightly higher among men [mean (standard deviation) Treatment Satisfaction Questionnaire for Medication-9 (TSQM-9) score: 68.6 (18.6) and 69.9 (18.2), respectively] than women [65.3 (18.2), 66.2 (18.5)].

***Conclusions:*** Overall response to biologics for PsA was rather favorable. With similar baseline disease severity, men appear to have a somewhat earlier and better response with higher treatment satisfaction.

## Introduction

Psoriatic disease is a systemic inflammatory disorder, including as main phenotypes psoriasis and psoriatic arthritis (PsA).^1,2^ PsA is a seronegative spondyloarthropathy characterized by the involvement of peripheral joints, the axial skeleton, entheses, skin, and nails.^3,4^ Although men and women are rather equally affected with PsA, phenotypic features and outcome differences between sexes have been reported. Women have been reported to have more frequently polyarthritis,^5–9^ elevated inflammatory markers,^8,10^ more erosive disease,^11^ worse pain^7,8,12^ and fatigue,^6,8,12,13^ more functional limitation and work disability,^7,8,11,13^ and worse quality of life.^6^ On the other hand, men seem to have more frequently axial disease,^5–7,10^ nail psoriasis,^6^ more severe skin involvement,^8^ and more rapid radiographic progression.^6,11^ In terms of treatment outcomes, in general, men have been reported to achieve remission or minimal disease activity more often than women.^7^ Focusing on biologic agents, effectiveness of TNF-inhibitors (TNFis) was reported to be higher in men,^7,10,13^ as was persistence in treatment.^10,14,15^ However, evidence of overall effectiveness of biologics in the real-world practice by gender is still scarce.

CHRONOS (EffeCtiveness of biologic treatments for PsA in Italy: An ObservatioNal lOngitudinal Study of real-life clinical practice) was a multicenter, real-world study, conducted in 20 Italian hospital rheumatology clinics in adult patients with PsA treated with a biologic medication. The aim of the CHRONOS was to provide real-world evidence of the effectiveness of biologic treatments for PsA in the Italian real-life clinical practice, primarily in terms of clinical response to the study biologic therapy at 6 months and 1 year after treatment initiation according to the EULAR Disease Activity Score 28 joints (DAS28) response criteria. A secondary objective of the study was to describe the treatment response, the withdrawal rates from study biologic treatments, the proportion of patients switching from one biologic treatment to another, and the patient's treatment satisfaction from a gender perspective.

This gender analysis is part of the larger MetaGeM project that Novartis has put in place since some years, aimed at evaluating gender differences occurring in therapeutic approaches, clinical outcomes, and safety parameters, by means of *post hoc* analyses and meta-analyses of previously conducted observational trials.^16–23^ The MetaGeM project includes so far 12 Italian observational studies in different clinical areas, encompassing immune-mediated disorders, organ transplants, and infectious, central nervous system, cardiovascular, and respiratory diseases, performed between 2002 and 2020. We report, in this study, the results of the CHRONOS gender analysis.

## Patients and Methods

### Study design and participants

CHRONOS was a multicenter, noninterventional, longitudinal cohort study involving both retrospective and prospective data. The study was conducted in 20 Italian hospital rheumatology clinics. Patients ≥18 years of age, with diagnosis of PsA made by a rheumatologist, providing written informed consent before the data collection, who had initiated a biologic treatment between 24 weeks and 24 months before enrollment visit (retrospective period), were consecutively enrolled when spontaneously referring to the clinic according to ordinary clinical practice, provided they had available data for DAS28 in the retrospective period. Patients who had interrupted treatment before enrollment could also be included. Stopping biologic treatment was not a reason for study exit. Detailed description of eligibility and evaluability criteria are reported elsewhere.^24^ The study was approved by the Ethic Committee of the coordinating center (A.O.U. Policlinico-Vittorio Emanuele P.O. San Marco, Catania, Italy).

Retrospective data were collected back at enrollment visit from hospital medical charts or other clinical documents, whereas prospective data were collected at the enrollment and 6-month (±1 month) follow-up visits, which took place as per normal clinical practice.

### Outcome measures

The primary outcome of the study was the proportion of patients with PsA achieving clinical response to the biologic therapy ongoing at enrollment or taken last before the enrollment visit, in case the patient was no longer assuming a biologic at enrollment (henceforth referred to as “biologic treatment under analysis”), measured 6 months and 1 year after treatment initiation by the EULAR DAS28 response criteria.^25^

Moreover, as sensitivity analysis, the proportion of patients achieving response at 6 months and 1 year after treatment initiation was calculated, where available in the medical charts, according to the American College of Rheumatology (ACR) criteria. Obtaining ACR20 means achieving 20% improvement in tender or swollen joint counts as well as a 20% improvement in at least three out of the other five criteria (patient assessment, physician assessment, pain scale, disability/functional questionnaire, erythrocyte sedimentation rate [ESR], or C-reactive protein [CRP]). Similar is the interpretation for ACR50 and ACR70.^26^

The clinical response was evaluated also in terms of patients with dactylitis (the presence and number of affected fingers), enthesitis (the presence and location evaluated by Leed Enthesitis Index [LEI]), and the presence of axial arthritis (according to the physician) over the study observation period.

Secondary outcome measures were the psoriasis skin burden and severity, evaluated by means of the Psoriasis Area and Severity Index (PASI) score—ranging 0–72 and increasing with increasing severity—, the patients' functional status, measured by the Health Assessment Questionnaire Disability Index (HAQ-DI)—ranging 0–3 and increasing with increasing disability,^27^—and patient's treatment satisfaction, assessed at enrollment and follow-up visits by means of the Treatment Satisfaction Questionnaire for Medication-9 (TSQM-9) subscale scores.^28,29^ Switchers were defined as patients switching from a branded/biosimilar to a branded/biosimilar of another class (changes in dosage or frequency within the same therapy class did not configure a switch); discontinuing patients were not-switchers who interrupted biologic treatment under analysis before the end of observation.

### Sample size

According to feasibility considerations, assuming the inclusion in the CHRONOS study of 400 patients affected by PsA (15% of whom could have been not available for the primary analysis), we simulated on 340 patients the achievable precision of the primary endpoint at 6 months and 1 year after initiation of the biologic treatment under analysis. The reached sample size of patients evaluable for the primary objective, even if lower than 340, allowed precise estimates of the primary endpoints, as detailed in the CHRONOS main article.^24^ This being a *post hoc* analysis, no sample size justification was performed for the gender analysis presented in this study.

### Statistical analyses

No formal statistical hypotheses were set. This is a descriptive study and was not powered to detect a difference in gender outcomes. Descriptive analyses were performed overall and stratified by gender; quantitative variables were described by mean, standard deviation (SD), median, 25th and 75th percentile, minimum, and maximum, whereas qualitative variables by absolute and relative frequency. Patients violating study criteria were excluded. Patients with follow-up visits performed outside the tolerance window defined by study protocol were not excluded.

DAS28 was calculated, based on CRP and on ESR,^30^ at start of the biologic treatment under analysis and after 6 months and 1 year. Response rates were calculated at each time point (6 months, 1 year) as the ratio between number of responders (*i.e.*, patients achieving a good or moderate response according to EULAR DAS28 response criteria) and number of patients with available information on achievement or nonachievement of response.

At each study visit, TSQM-9 questionnaire subscores (effectiveness, convenience, and global satisfaction), ranging from 0 to 100 with higher scores representing higher satisfaction, were calculated by applying instructions provided by the authors.^28,29^

Site monitoring, data management, and statistical analysis were performed by MediNeos (Modena, Italy). Database management and data analysis were performed using SAS Enterprise Guide v. 7.1 and SAS 9.4.

## Results

Overall, 409 patients were enrolled in the CHRONOS between September 2018 and September 2019. Following the exclusion of 10 patients who did not meet the inclusion criteria, the eligible patients remained 399 (97.6%). Men were 43.1% (*N* = 172). The sociodemographic and baseline main clinical characteristics by gender are reported in Table 1. The percentage of current and previous smokers was overall fairly high and slightly higher in women (84.2%) than in men (78.7%). Overweight patients were more among men than among women (*N* = 66, 49.3% vs. *N* = 48, 27.1%). In men, duration of psoriasis (17.3, SD 11.9) and PsA (8.6, SD 8.3) were somewhat higher than in women (13.3, SD 12.3, and 6.1, SD 5.5, respectively). The type of arthritis at baseline by gender is summarized in Table 2. Among eligible patients, 66.1% of men and 57.3% of women had comorbidities at enrollment, the most common being hypertension (39.8% of men, *N* = 68 and 26.0% of women, *N* = 59), followed by diabetes (12.3%, *N* = 21 and 7.5%, *N* = 17, respectively), hypercholesterolemia/dyslipidemia (9.4%, *N* = 16 and 9.3%, *N* = 21, respectively), and other autoimmune diseases (4.1%, *N* = 7 and 6.2%, *N* = 14, respectively).

**Table 1.** Demographic and Baseline Main Clinical Characteristics by Gender

	Men^a^	Women^a^
Age at enrollment, years, Mean (SD)	*172*	*227*
	51.9 (11.7)	52.7 (11.4)
Smoking status at enrollment, *n* (%)	*145*	*203*
Nonsmoker	31 (21.4)	32 (15.8)
Current smoker	91 (62.8)	145 (71.4)
Previous smoker	23 (15.9)	26 (12.8)
BMI classes at enrollment, *n* (%)	*134*	*177*
Underweight (<18.5)	37 (27.6)	40 (22.6)
Normal weight ([18.5–25[)	28 (20.9)	83 (46.9)
Overweight ([25–30])	66 (49.3)	48 (27.1)
Obese (≥30)	3 (2.2)	6 (3.4)
Duration of psoriasis, years, Mean (SD)	*112*	*114*
	17.3 (11.9)	13.3 (12.3)
Duration of psoriatic arthritis, years, Mean (SD)	*169*	*223*
	8.6 (8.3)	6.1 (5.5)
DAS28 ESR at start of study biologic treatment, Mean (SD)	*119*	*160*
	3.7 (1.3)	4.2 (1.3)
DAS28 CRP at start of study biologic treatment, Mean (SD)	*139*	*173*
	3.7 (1.2)	3.8 (1.2)
Duration of biologic treatment under analysis (months), Mean (SD)	*172* 18.3 (6.7)	*227* 18.9 (6.4)
No. of biologic therapies received before biologic treatment under analysis, *n* (%)	*172*	227
0	84 (48.8)	102 (44.9)
1	56 (32.6)	77 (33.9)
2	19 (11.0)	21 (9.3)
≥3	13 (7.5)	27 (11.9)
No. of biological therapies received during study, *n* (%)	*172*	*227*
1	145 (84.3)	178 (78.4)
2	23 (13.4)	34 (15.0)
3–4	4 (2.4)	15 (6.6)
Patients with dactylitis, *n* (%)	*73*	*75*
	16 (21.9)	19 (25.3)
Patients with enthesitis, *n* (%)	*71*	*76*
	21 (29.6)	28 (36.8)
Patients with axial arthritis, *n* (%)	*48*	*52*
	28 (57.1)	30 (56.6)

All percentages are calculated on the number of patients with available data.

^a^ No. of patients with available data is showed in italics.

CRP, C-reactive protein; DAS28, Disease Activity Score 28 joints; ESR, erythrocyte sedimentation rate; SD, standard deviation.

**Table 2.** Type of Arthritis at Enrollment by Gender

Type of arthritis	Men *n* (%)	Women *n* (%)
Asymmetric oligoarticular	65 (37.8)	90 (39.6)
Symmetric polyarthritis	79 (45.9)	99 (43.6)
Predominant distal interphalangeal	11 (6.4)	8 (3.5)
Spondylitis	32 (18.6)	49 (21.6)
Arthritis mutilans	1 (0.6)	2 (0.9)

Data were available for all eligible patients, 172 men and 227 women.

The proportion of patients naive to biologic treatment before study treatment were 44.9% (*N* = 102) among women and 48.8% (*N* = 84) among men. One or two previous biologic medications had been assumed by 43.2% (*N* = 98) of women and 43.6% (*N* = 75) of men, whereas 11.9% (*N* = 27) of women and 7.5% (*N* = 13) of men had taken 3 or more (Table 1). The most frequently used among biologic treatments under analysis was secukinumab in both sexes (40.1% of women, *N* = 91 and 40.7% of men, *N* = 70), followed by adalimumab (including biosimilars, 17.2%, *N* = 39 and 18.6%, *N* = 32, respectively), and etanercept (including biosimilars, 15.9%, *N* = 36 and 17.4%, *N* = 30, respectively) (Table 3). However, taken together, TNFis were received by 55.5% of women and 52.3% of men.

**Table 3.** Type of Biologic Treatment by Gender

	Men (*n* = 172)	Women (*n* = 227)
	*n* (%)	*n* (%)
Biological therapy: drug		
Secukinumab	70 (40.7%)	91 (40.1%)
Adalimumab (including biosimilars)	32 (18.6%)	39 (17.2%)
Etanercept (including biosimilars)	30 (17.4%)	36 (15.9%)
Certolizumab	11 (6.4%)	28 (12.3%)
Ustekinumab	12 (7.0%)	18 (7.9%)
Golimumab	10 (5.8)	10 (4.4%)
Infliximab (including biosimilars)	7 (4.1%)	5 (2.2%)

The mean total duration of the biologic treatment line under analysis was 18.3 (SD 6.7) months for men and 18.9 (6.4) months for women. Around 78.4% (*N* = 178) of women and 84.3% (*N* = 145) of men were treated with only one biologic during the study, 15.0% (*N* = 34) and 13.4% (*N* = 23), respectively, were treated with two, and 6.6% (*N* = 15) and 2.4% (*N* = 4), respectively, were treated with 3 or 4. Treatment discontinuations were slightly more common in men than women (6.4%, *N* = 11 vs. 3.5%, *N* = 8), whereas switches from one biological to another were more frequent in women than men (11.0%, *N* = 25 vs. 4.7%, *N* = 8).

Considering 176 females and 132 men with available response, DAS28 responders at 6 months were 72.7% among women and 70.5% among men. At 1 year, the overall proportion of patients achieving clinical response according to EULAR DAS28 criteria was 68.0% both among women and men (considering 169 women and 128 men with available response). In the subsample of CHRONOS patients with available ACR data, responses by gender at 6 months and 1 year are summarized in Table 4. The individual components of DAS28 and ACR response criteria at all study visits are reported in Table 5.

**Table 4.** Proportion of Patients Achieving Clinical Response at 6 Months and 1 Year (According to ACR20, ACR50, and ACR70 Criteria) by Gender

	Men *n* (%)	Women *n* (%)
6 months		
ACR20 response	*n* = 92 42.4%	*n* = 102 40.2%
ACR50 response	*n* = 92 32.6%	*n* = 102 26.5%
ACR70 response	*n* = 94 22.3%	*n* = 105 12.4%
1 year		
ACR20 response	*n* = 86 38.4%	*n* = 103 34.9%
ACR50 response	*n* = 86 34.9%	*n* = 105 20.0%
ACR70 response	*n* = 88 25.0%	*n* = 108 13.0%

Percentages computed on the number of patients reported above each percentage.

**Table 5.** Disease Activity Score 28 Joints (DAS28) and American College of Rheumatology (ACR) Components by Gender at Study Visits

	Index date		6 months		12 months	
	Males	Females	Males	Females	Males	Females
DAS28 components, mean (SD)						
Tender joint count (0–28)	*152* 4.1 (4.4)	*198* 5.3 (5.0)	*130* 1.0 (1.9)	*173* 2.6 (5.2)	*124* 1.1 (2.2)	*162* 1.6 (3.1)
Swollen joint count (0–28)	*152* 2.4 (3.0)	*198* 2.7 (3.3)	*130* 0.3 (0.8)	*173* 0.6 (1.5)	*124* 0.2 (0.6)	*162* 0.5 (1.3)
CRP (mg/L)	*139* 23.2 (58.4)	*173* 16.2 (37.9)	*120* 7.3 (15.7)	*157* 6.9 (11.8)	*115* 9.4 (19.6)	*147* 8.8 (23.7)
ESR (mm/hr)	*119* 20.6 (20.0)	*160* 24.1 (17.0)	*106* 10.4 (10.1)	*141* 18.9 (16.1)	*105* 12.0 (12.6)	*140* 19.1 (15.5)
General health patient (VAS 0–100 mm)	*130* 51.9 (23.0)	*153* 61.0 (22.2)	*106* 39.9 (29.7)	*120* 42.5 (20.6)	*104* 42.4 (30.4)	*121* 34.4 (22.8)
ACR components, mean (SD)						
Tender joint count	*141* 4.4 (4.6)	*144* 5.5 (6.3)	*91* 1.5 (3.1)	*97* 3.5 (6.7)	*81* 1.3 (2.2)	*89* 1.9 (4.1)
Swollen joint count	*141* 2.6 (3.2)	*144* 2.3 (3.2)	*91* 0.4 (1.2)	*97* 0.6 (1.6)	*81* 0.3 (0.7)	*89* 0.8 (3.2)
CRP (mg/L)	*138* 23.4 (58.6)	*173* 16.5 (37.9)	*81* 4.8 (7.1)	*82* 6.4 (11.3)	*69* 6.6 (13.8)	*78* 6.5 (10.9)
ESR (mm/hr)	*118* 20.6 (20.1)	*161* 24.0 (16.9)	*67* 11.1 (10.1)	*75* 17.3 (13.4)	*59* 13.5 (13.0)	*76* 18.6 (13.5)
Patient's assessment of pain (VAS 0–100 mm)	*123* 57.6 (25.1)	*117* 63.2 (22.8)	*86* 29.7 (25.2)	*78* 37.4 (25.0)	*77* 32.0 (28.1)	*77* 32.6 (26.5)
Patient's GA of disease activity (VAS 0–100 mm)	*94* 54.2 (24.4)	*103* 62.4 (21.7)	*82* 31.8 (25.9)	*74* 34.5 (22.3)	72 31.3 (27.3)	*74* 31.8 (24.2)
Physician's GA of disease activity (VAS 0–100 mm)	*88* 42.5 (25.7)	*88* 50.1 (24.0)	*76* 17.8 (21.4)	65 21.9 (19.9)	*66* 17.0 (20.5)	*71* 16.7 (18.1)
HAQ-DI (0–3)	*65* 1.0 (0.7)	*61* 1.0 (0.6)	*55* 0.7 (0.7)	*38* 0.9 (0.6)	*46* 0.5 (0.6)	44 0.7 (0.5)

All percentages are calculated on the number of patients with available data.

No. of patients with available data is shown in italics in the table.

ACR, American College of Rheumatology; GA, Global Assessment; HAQ-DI, Health Assessment Questionnaire Disability Index; VAS, visual analogue scale.

Considering some specific clinical characteristics of PsA, at 6 months, patients with dactylitis (*n/N* = 19/75 women and *n/N* = 16/73 men) decreased from 25.3% to 2.8% (*n/N* = 2/72) in women and from 21.9% to 4.3% (*n/N* = 3/70) in men; patients with enthesitis (*n/N* = 28/76 women and 21/71 men) decreased from 36.8% to 4.3% (*n/N* = 3/69) in women and from 29.6% to 14.1% (*n/N* = 9/64) in men; patients with axial arthritis (*n/N* = 30/52 women and 28/48 men) decreased from 57.7% to 54.8% (*n/N* = 23/42) in women and from 58.3% to 43.6% (*n/N* = 17/39) in men. Further changes in these three clinical aspects between 6 months and 1 year were modest.

**FIG. 1.**
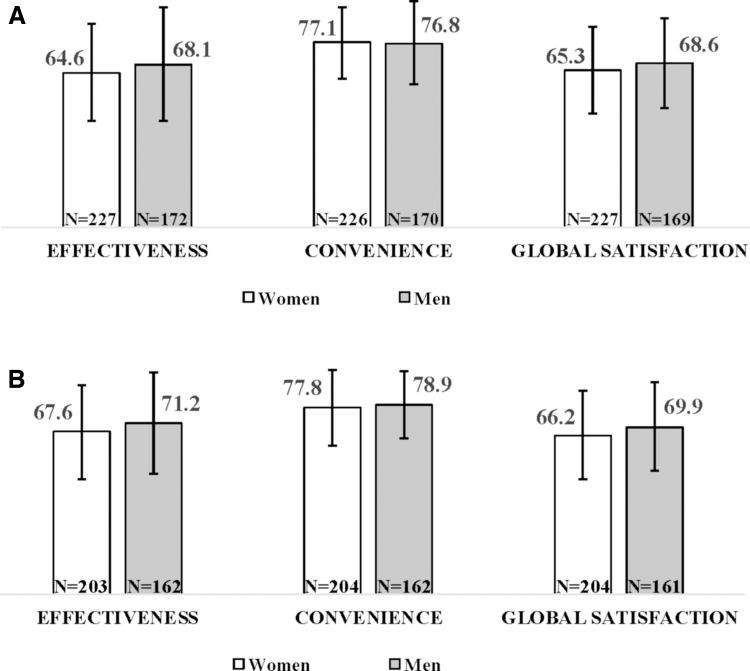
Patients' satisfaction with biologic treatment (measured by TSQM-9 subscales) by gender at **(A)** enrollment and **(B)** 6 months follow-up. TSQM-9, Treatment Satisfaction Questionnaire for Medication-9.

In terms of psoriatic skin lesions, men had a higher mean PASI (4.0, SD 4.8; *N* = 46) than women (2.4, SD 5.3; *N* = 41) at start of therapy. At 6 months, the mean PASI had decreased to 0.3 (SD 0.6) in the female population (*N* = 41) and to 0.8 (SD 1.7) in the male population (*N* = 46). At 1 year, mean PASI scores remained substantially unchanged compared with 6-month values in both men and women (0.9, SD 1.4 and 0.3, SD 0.7, respectively). Disability was mild/moderate at start of therapy in the small number of patients with available data, with mean HAQ-DI of 1.0 (SD 0.5) in women (*N* = 30) and 0.9 (SD 0.6) among men (*N* = 35). Values decreased after 6 months of biological therapy to 0.8 (SD 0.5) and 0.6 (SD 0.6), respectively, and remained unchanged at 1 year. Patients' global satisfaction with biological treatments, measured by TSQM-9 global satisfaction subscale, was slightly higher among men than among women, both at enrollment and after 6 months of treatment with biologic agents. The trend for the three TSQM-9 subscales at the two study time points is illustrated in Figure 1.

## Discussion

The CHRONOS patients were enrolled in different Italian hospital rheumatology clinics according to wide and simple inclusion/exclusion criteria to reflect as much as possible the PsA population observed in the clinical practice. Women were slightly more than men, but overall, the two genders were rather well balanced for demographic and baseline clinical characteristics. It may be observed that more men were overweight or obese, whereas, consistently with the latest smoking trends, there were slightly more current smokers among women. The duration of the disease was little longer in men, especially for psoriasis, whereas the location/type of arthritis were rather homogeneous in both sexes.

Comorbidities were present in more than half of patients of both sexes, with hypertension and diabetes, which often go together, apparently more common in men, and autoimmune diseases—other than PsA—slightly more frequent in women, consistently with the knowledge that females are generally more frequently affected with autoimmune diseases than males, possibly due to the impact of sexual hormones.^31,32^ No marked differences were observed in the proportion of patients naive to biologics before study treatment and in the number of biologic treatment lines prescribed before study treatment.

The most used study biologic medications were TNFis, mainly split fairly evenly between adalimumab and etanercept, and secukinumab, with no differences between sexes. The mean duration of the study biologic treatment was similar in men and women with little more women than men treated with more than one single drug during the study. Consistently, the frequency of switches from one biological to another was higher in women.

In terms of clinical response, a very recent article on clinical gender differences in PsA reported that male patients had significantly higher remission rates.^33^ Conversely, in our analysis, the proportion of DAS28 responders at 6 months and 1 year was homogeneous between sexes; however, men seemed to respond earlier, given that at 6 months more men had a good response, whereas women had more moderate responses. ACR response data were available in a lower number of patients, however, there was a trend for men to achieve more frequently the ACR50/70 targets at both study time points. Looking at the individual components of DAS28 and ACR it is worth underlining that the subjective components of both indexes are higher in women than in men, especially at enrollment, still somewhat at 6 months, while they tend to equal at 12 months. With a similar mean of swollen joints, the mean of painful joints was higher in women and the evaluation of the general health status was worse in women as was the patient's assessment of pain.

This is consistent with literature data strongly suggesting that men and women differ in their sensitivity and responses to pain. Pain sensitivity is increased in women and pain and painful diseases are more commonly reported by women; moreover, response to pain reliever is much more variable in women.^34,35^ It has been discussed that not only social and psychological factors play a role in such differences, but also biological differences in the functioning of the immune system and related to sex hormones are likely to be involved. Among objective components, women had consistently higher ESR mean values, but not CRP, compared with men. Previous studies demonstrated that there is discordance between DAS28-ESR and DAS28-CRP, with DAS28-CRP tending to yield lower values of disease activity than DAS28-ESR,^36^ and that discordance is greatest for women.^37^ Treatment response was marked for dactylitis already at 6 months in both sexes; it was also particularly notable for enthesitis in women, somewhat less in men. On the other hand, response in terms of axial spondylitis was absent in women and modest in men.

Published evidence suggests there can be anatomical and immunological differences between axial and peripheral enthesitis, and downstream disease manifestations and discrepant responses to IL-17A inhibition are observed in spondylarthritis manifestations.^38^ Furthermore, studies analyzing gender differences in axial spondylitis revealed that female patients have different disease manifestations due to different immunological, hormonal, and genetic responses, with an overall higher disease activity and a lower quality of life. Moreover, women were reported to have lower response to TNFis and lower drug adherence.^39,40^

Psoriatic skin lesions that were already mild at the start of the study, as they had probably already been treated previously, practically disappeared already at 6 months. Disability, measured by HAQ-DI, changed truly little during the study in both sexes, but it has to be noted that it was already low at the beginning of the study. The level of satisfaction with treatment was slightly lower in women, both at enrollment and after 6 months, and this is consistent with the higher rate of changes between biologics before and during the study.

Taken altogether, the results of the CHRONOS study analyzed by gender give us the picture of a real-life PsA population that is quite homogeneous between sexes. We did not observe, in our cohort, different disease characteristics as reported in the literature:^5–9^ men and women had fairly the same patterns of arthritis, as well as level of axial involvement, and disability. The clinical response to biologicals was rather favorable in both sexes. However, with similar baseline disease severity, men showed an earlier and higher quality overall with clinical response to biologics, in line with what was previously reported.^7,10,13^ This could explain why the women of our cohort were less satisfied with treatment and changed the biologic medication more frequently.

The CHRONOS study had some limitations, mainly due to its observational and real-life nature, such as the heterogeneity of patients in terms of clinical history and previous treatments for PsA, the great variability in the number of patients with available data for different measures and at different time points, and also some inhomogeneity in the quality of the collected data, due to the retrospective and prospective phases of the study. We required in the protocol that patients had a diagnosis of PsA according to the investigators' judgment; a postenrollment check showed that not all enrolled patients completely fulfilled CASPAR criteria, however, given that CASPAR criteria were not specified among inclusion criteria, we included in the analysis all patients diagnosed by their rheumatologist. Another limitation is that only rheumatoid arthritis-specific outcome measures were used (ACR and DAS), which we know are suboptimal for outcome assessment in PsA.

This choice was due to the real-life nature of the study, given that Italian hospital rheumatologists routinely perform ACR and DAS, rather than PASDAS (Psoriatic Arthritis Disease Activity Score) or DAPSA (Disease Activity in PSoriatic Arthritis). This analysis has the further limitation that the study was not powered to detect differences between genders, since this was a secondary objective of the CHRONOS study. On the other hand, the study provides descriptive information on real-world data, about the typical PsA patients accessing hospital rheumatology clinics in Italy.

## Conclusions

This gender analysis of the CHRONOS adds to the other numerous gender *post hoc* analyses of the MetaGeM project—an Italian gender medicine program that has evaluated gender differences in several observational real-life studies conducted in different therapeutic areas^16–23^—confirming that some differences exist between men and women in the clinical response to and perception of both disease and treatments, thus suggesting once more that gender attention may be required in view to provide tailored therapeutic options.

## Ethics Approval

The study was conducted in accordance with the amended Declaration of Helsinki.

## Members of the CHRONOS Study Group—Participating Centers

Frassi Micol (ASST Spedali Civili, Brescia); Caminiti Maurizio (Grande Ospedale Metropolitano Bianchi Melacrino Morelli, Reggio Calabria); Fusaro Enrico (AOU Città della Salute e della Scienza di Torino, Turin); Lomater Claudia (A.O. Mauriziano, Turin); Del Medico Patrizia (Ospedale civile, Civitanova Marche); Iannone Fiorenzo (A.O.U. Policlinico Consorziale, Bari); Foti Rosario (A.O.U. Policlinico—Vittorio Emanuele, Catania); Limonta Massimiliano (ASST Papa Giovanni XXIII, Bergamo); Marchesoni Antonio (Department of Rheumatology, ASST Gaetano Pini-CTO, Milan); Raffeiner Bernd (Ospedale Centrale di Bolzano, Bolzano); Viapiana Ombretta (AOUI Verona Borgo Roma, Verona); Grassi Walter (Policlinico A. Murri, Jesi); Grembiale Rosa Daniela (A.O.U. Mater Domini, Catanzaro); Guggino Giuliana (A.O.U. Policlinico Giaccone, Palermo); Mazzone Antonino (Ospedale Civile di Legnano, Legnano); Tirri Enrico (Ospedale San Giovanni Bosco, Naples); Perricone Roberto (Policlinico Tor Vergata, Rome); Sarzi Puttini Pier Carlo (ASST FBF Sacco, Milan); De Vita Salvatore (ASUIUD, Udine); and Conti Fabrizio (Azienda Policlinico Umberto I, Rome).

## Notes

**Cite this article as:** Colombo D, Frassi M, Pagano Mariano G, Fusaro E, Lomater C, Del Medico P, Iannone F, Foti R, Limonta M, Marchesoni A, Raffeiner B, Viapiana O, Di Carlo M, Grembiale RD, Guggino G, Faggioli P, Tirri E, Perricone R, Sarzi Puttini PC, De Vita S, Conti F, Rizzoli S, Roncari B, Fiocchi M, Orsenigo R, Zagni E (2022) The CHRONOS real-world evidence of biologic treatments in psoriatic arthritis in Italy: A *post hoc* gender analysis, *Women's Health Report* 3:1, 162–171, DOI: 10.1089/whr.2021.0063.

## Abbreviations Used

ACR: American College of Rheumatology
CRP: C-reactive protein
DAPSA: Disease Activity in PSoriatic Arthritis
DAS28: Disease Activity Score 28 joints
ESR: erythrocyte sedimentation rate
GA: Global Assessment
HAQ-DI: Health Assessment Questionnaire Disability Index
LEI: Leed Enthesitis Index
PASDAS: Psoriatic Arthritis Disease Activity Score
PASI: Psoriasis Area and Severity Index
PsA: psoriatic arthritis
SD: standard deviation
TNF: tumor necrosis factor
TNFis: TNF-inhibitors
TSQM-9: Treatment Satisfaction Questionnaire for Medication-9
VAS: visual analogue scale
